# Author Correction: Inhibition of a nutritional endosymbiont by glyphosate abolishes mutualistic benefit on cuticle synthesis in *Oryzaephilus surinamensis*

**DOI:** 10.1038/s42003-021-02614-z

**Published:** 2021-09-09

**Authors:** Julian Simon Thilo Kiefer, Suvdanselengee Batsukh, Eugen Bauer, Bin Hirota, Benjamin Weiss, Jürgen C. Wierz, Takema Fukatsu, Martin Kaltenpoth, Tobias Engl

**Affiliations:** 1 Evolutionary Ecology, Institute of Organismic and Molecular Evolution (iomE), Johannes Gutenberg University, Mainz, Germany; 2 Bioproduction Research Institute, National Institute of Advanced Industrial Science and Technology (AIST), Tsukuba, Japan; 3 Department of Biological Sciences, Graduate School of Science, University of Tokyo, Tokyo, Japan; 4 Research Group Insect Symbiosis, Max-Planck-Institute for Chemical Ecology, Jena, Germany; 5 Graduate School of Life and Environmental Sciences, University of Tsukuba, Tsukuba, Japan; 6 Department of Insect Symbiosis, Max-Planck-Institute for Chemical Ecology, Jena, Germany

**Keywords:** Chemical ecology, Evolutionary ecology

Correction to: *Communications Biology* 10.1038/s42003-021-02057-6, published online 11 May 2021.

The original version of the Article included an error in reporting the glyphosate concentration used in previous work. The “Glyphosate and aromatic amino acid supplementation” section of the methods incorrectly stated a previously reported value of 250 mg glyphosate applied per 48L of soil^90^ as 250 g glyphosate per 48L of soil, leading to the overestimation of the concentration as 0.4% instead of the correct 0.0004%. This sentence should read “Helander et al. report 250 mg per 48 L of soil to be equivalent to the maximum recommended amount of glyphosate for agronomical applications which translates to 0.0004% based on a fertile soil density of 1.3 g/cm³^90^.” This has been corrected in both the PDF and HTML versions of the Article.
