# Supplementary material for: Inhibition of a nutritional endosymbiont by glyphosate abolishes mutualistic benefit on cuticle synthesis in Oryzaephilus surinamensis
Source: Commun Biol. 2021 May 11;4:554. doi: 10.1038/s42003-021-02057-6 (PMC8113238; doi:10.1038/s42003-021-02057-6)
Supplement: Supplementary file 1 — Supplementary Information [file 42003_2021_2057_MOESM1_ESM.pdf]

Supplementary Information from: Inhibition of a nutritional endosymbiont by glyphosate abolishes mutualistic benefit on cuticle synthesis in *Oryzaephilus surinamensis*

Julian Simon Thilo Kiefer[1], Suvdanselengee Batsukh[1], Eugen Bauer[1], Bin Hirota[2,3], Benjamin Weiss[1,5], Jürgen C. Wierz[1], Takema Fukatsu[2,3,4], Martin Kaltenpoth[1,5,6], Tobias Engl[1,5,6*]

[1] Evolutionary Ecology, Institute of Organismic and Molecular Evolution (iomE), Johannes Gutenberg 9

[2] Bioproduction Research Institute, National Institute of Advanced Industrial Science and Technology (AIST), Tsukuba 305-8566, Japan

[3] Department of Biological Sciences, Graduate School of Science, University of Tokyo, Tokyo 113-0033, Japan

[4] Graduate School of Life and Environmental Sciences, University of Tsukuba, Tsukuba 305-8571, Japan

[5] Research Group Insect Symbiosis, Max-Planck-Institute for Chemical Ecology, Hans-Knoell-Straße 8, 07745 Jena, Germany

[6] Department of Insect Symbiosis, Max-Planck-Institute for Chemical Ecology, Hans-Knoell-Straße 8, 07745 Jena, Germany

[*] Corresponding author: tengl@ice.mpg.de, Max Planck Institute for Chemical Ecology, Department of Insect Symbiosis, Hans-Knoell-Str. 8, 07745 Jena, Germany, +49 6131 57 23572

Supplementary Table 1. General features of the *S. silvanidophilus* genome in comparison with genomes of Bacteroidetes symbionts of other insects. All genomes where re-annotated with RAST[1,2,3] and Prokka[4] in KBase[5]. The completeness of the single genomes was calculated with CheckM, which is using collocated sets of genes that are ubiquitous and single-copy within a phylogenetic lineage[6]. Note that this often strongly underestimates true genome completeness for obligate endosymbionts, because they tend to lose many core genes due to genome erosion.

Bacterium	*Walczuchella monophlebidarum* FNIIJ	*Uzinura diaspidicola* ASNER	*Blattabacterium celvelandi* CCLhc	*Shikimatogenerans silvanidophilus* OSUR	*Sulcia muelleri* OLIH	*Sulcia muelleri* PSPU
Host	Hemiptera *Llaveia axin axin*	Hemiptera *Aspidiotus nerii*	Isoptera *Mastotermes darwiniensis*	Coleoptera *Oryzaephilus surinamensis*	Hemiptera *Oliarus filicicola*	Hemiptera *Philaenus spumarius*
Accession	CP006873	CP003263	CP029844	JADFUB000000000	CP028359	AP013293
Genome Size	309,299 bp	263,431 bp	617,422 bp	307,680 bp	156,578 bp	285,352 bp
Plasmids	0	0	1 (3.3 kb)	0	0	0
Completeness	67.38 %	55.75 %	98.17 %	66.68 %	41.98 %	54.2 %
GC content	32.7 %	30.2 %	24.6 %	16.2 %	24.9 %	20.9 %
Predicted proteins	316	254	587	299	155	261
Ribosomal RNAs	3	3	3	3	3	3
SSU ribosomal proteins	21	20	21	21	20	21
LSU ribosomal proteins	32	31	31	30	26	30
Transfer RNAs	33	31	31	28	29	30

Supplementary Table 2. Aminoacyl tRNA synthetases and tRNAs encoded by the *S. silvanidophilus* genome in comparison with other Bacteroidetes insect symbiont genomes. Three letter entries indicate the presence of the tRNA anticodons, highlighted cells in blue indicate the presence of the corresponding aminoacyl tRNA synthetases.

Aminoacyl tRNA synthetase	*Walczuchella monophlebidarum* FNIIJ	*Uzinura diaspidicola* ASNER	*Blattabacterium celvelandi* CCLhc	*Shikimatogenerans silvanidophilus* OSUR	*Sulcia muelleri* OLIH	*Sulcia muelleri* PSPU
Ala	TGC, GGC	TGC	TGC	TGC	TGC	TGC
Arg	TCT, CCG, ACG	TCT, ACG	TCT, CCT, ACG	TCT, CCT, ACG	TCT, ACG	TCT, ACG
Asn	GTT	GTT	GTT	GTT	GTT	GTT
Asp	GTC	GTC	GTC	GTC	GTC	GTC
Cys	GCA	GCA	GCA	GCA	GCA	GCA
Gln	TTG	TTG	TTG	TTG	TTG	TTG
Glu	TTC	TTC	TTC	TTC	TTC	TTC
Gly	TCC, GCC	TCC, GCC	TCC, GCC	TCC	TCC, GCC	TCC, GCC
His	GTG	GTG	GTG	GTG	GTG	GTG
Ile	GAT	GAT	GAT	GAT	GAT	GAT
Leu	TAG, TAA, CAA	TAG,TAA, GAG, CAA	TAG, GAG, CAA	TAG, CAA	TAG, TAA	TAG, TAA, GAG
Lys	TTT	TTT	TTT	TTT	TTT	TTT
Met	CAT (3)	CAT (3)	CAT (3)	CAT (3)	CAT (3)	CAT (3)
Phe	GAA	GAA	GAA	GAA	GAA	GAA
Pro	TGG, GGG	TGG	TGG	TGG	TGG	TGG
Ser	TGA, GCT	TGA, GCT	TGA, GGA, GCT	TGA,GGA,GCT	TGA, GCT	TGA, GGA, GCT
Thr	TGT, GGT	TGT, GGT	TGT, GGT	TGT, GGT	TGT, GGT	TGT, GGT
Trp	CCA	CCA	CCA	CCA	CCA	CCA
Tyr	GTA	GTA	GTA	GTA	GTA	GTA, GAC
Val	TAC, GAC	TAC, GAC	TAC, GAC	TAC	TAC	TAC, GAC
Pseudo	AAG	CCG			GAG, CAA	

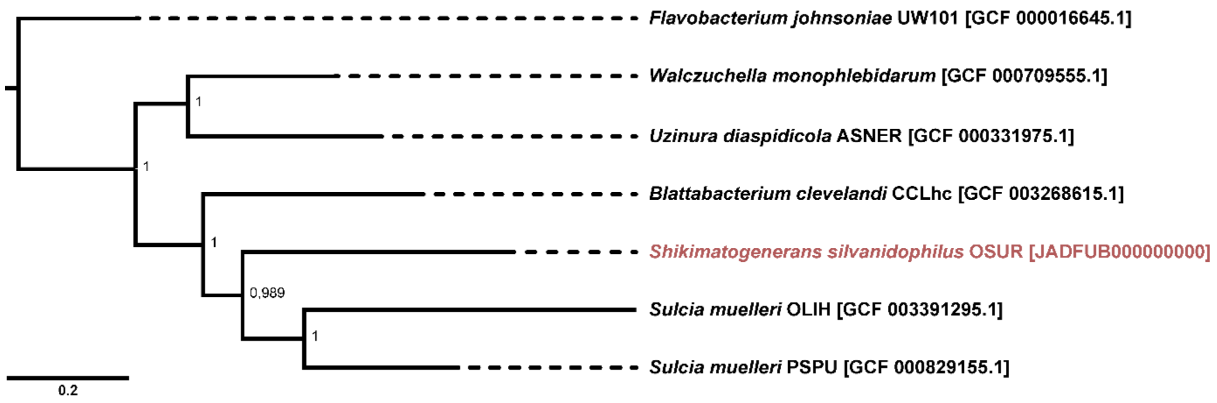

Supplementary Figure 1. Phylogenetic tree for the placement of the intracellular symbiont *S. silvanidophilus* in *O. surinamensis* within the Bacteroidetes, based on a defined list of 49 orthologous genes. The phylogeny was reconstructed using the KBase app *Insert Set of Genomes Into Species Tree* v2.2.0, based on FastTree2 algorithm[7]. Node numbers represent local support values. RefSeq assembly accession in square brackets.

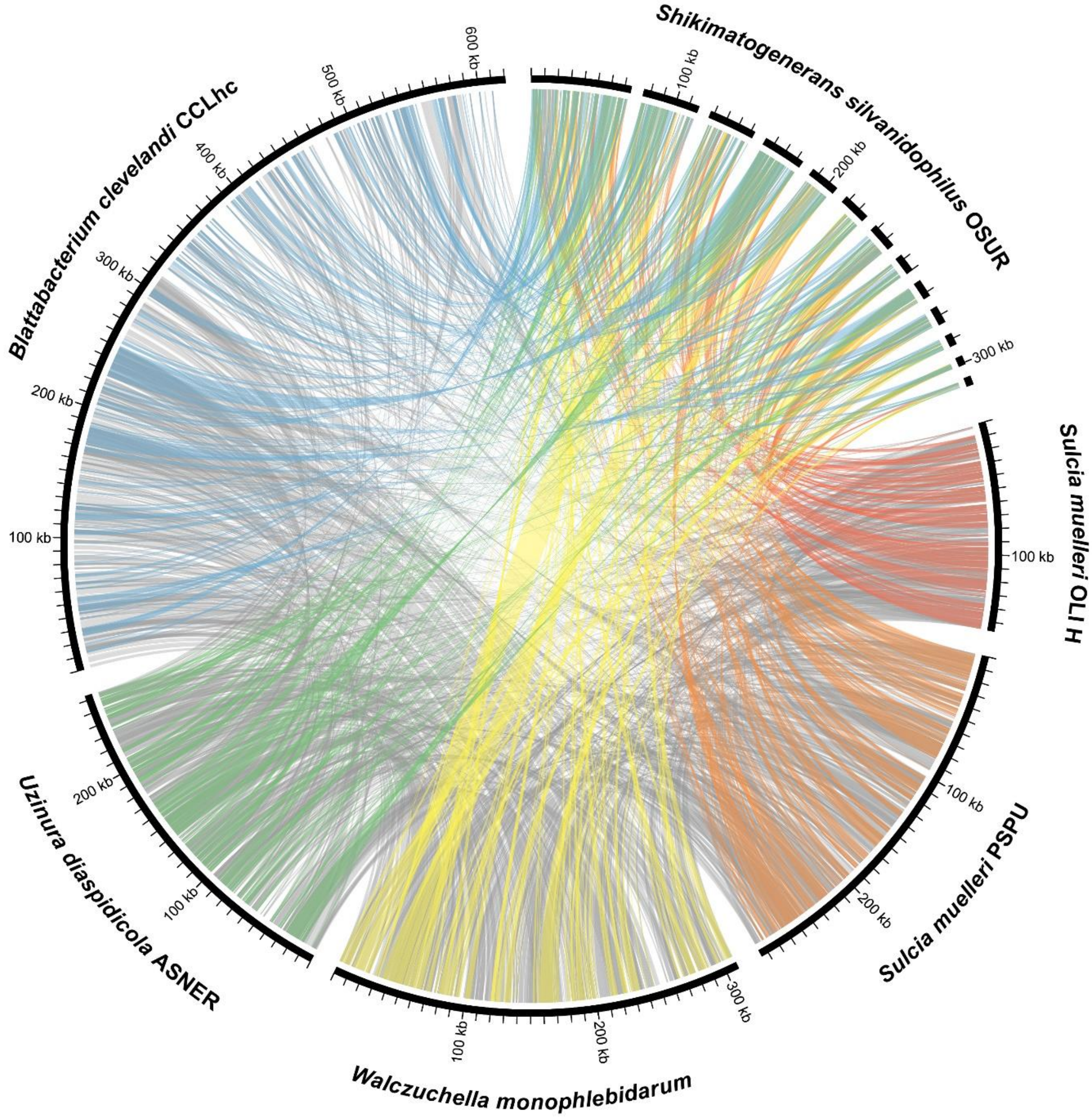

Supplementary Figure 2. Comparison of the functional gene repertoires of the endosymbiont and other Bacteroidetes symbionts in insects. Color: orthologs between *S. silvanidophilus* and another genome. Grey: Pairwise orthologs between the other Bacteroidetes insect symbionts.

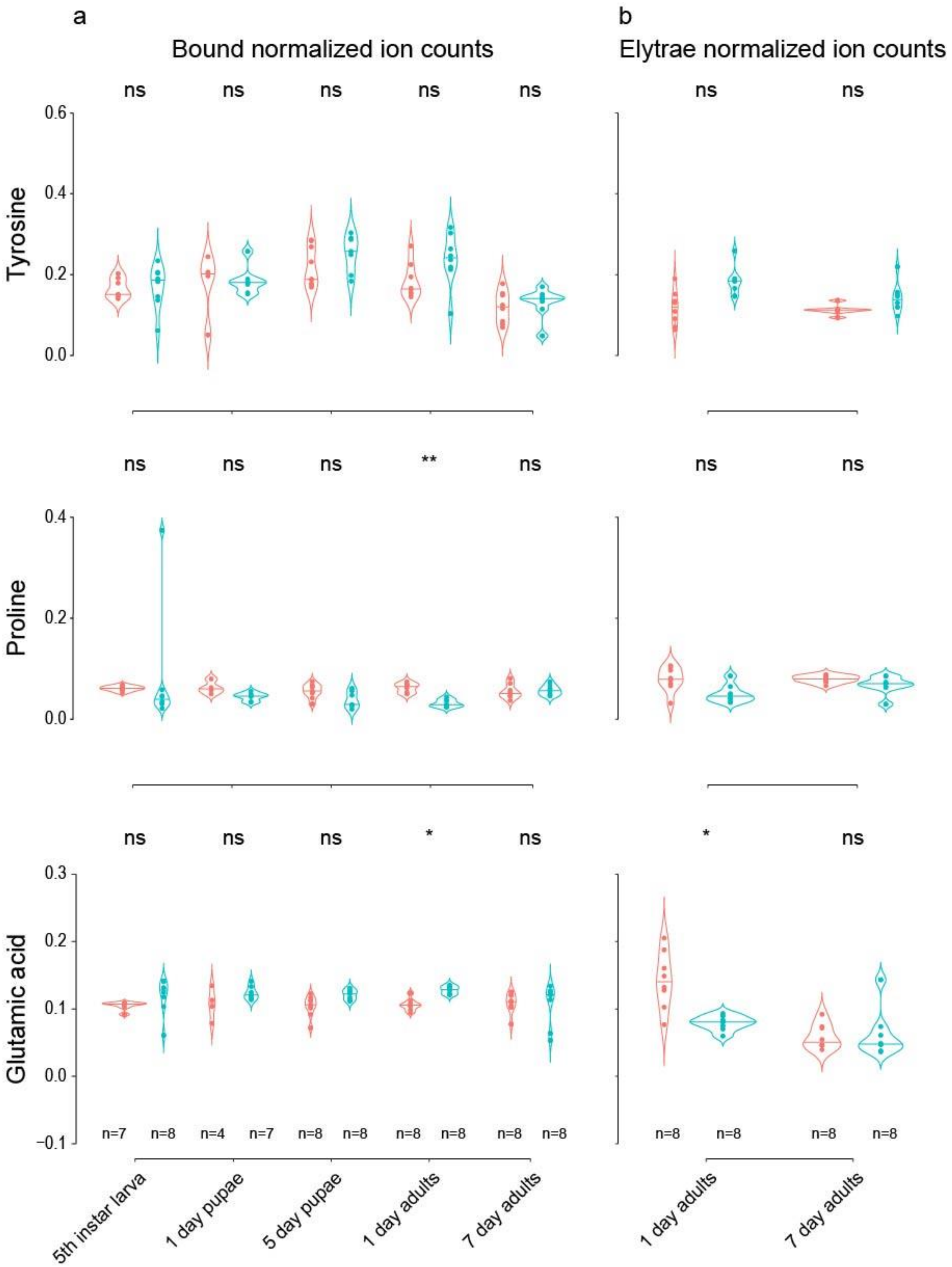

Supplementary Figure 3. Comparison of titers of the three amino acid tyrosine, proline and glutamic acid that that were influenced by symbiont presence. Shown are (a) bound amino acid titers in the whole body (without elytrae in case of adults) and (b) combined free and bound titers in adult elytrae of symbiotic and aposymbiotic *O. surinamensis* beetles. Red: aposymbiotic beetles, Blue: symbiotic beetles. The data distribution is visualized with violin plots and an additional horizontal line depicting the median. FDR corrected unpaired, two-sided Wilcox-rank-sum-tests: ns $p > 0.05$, * $0.05 < p < 0.01$, ** $p < 0.01$.

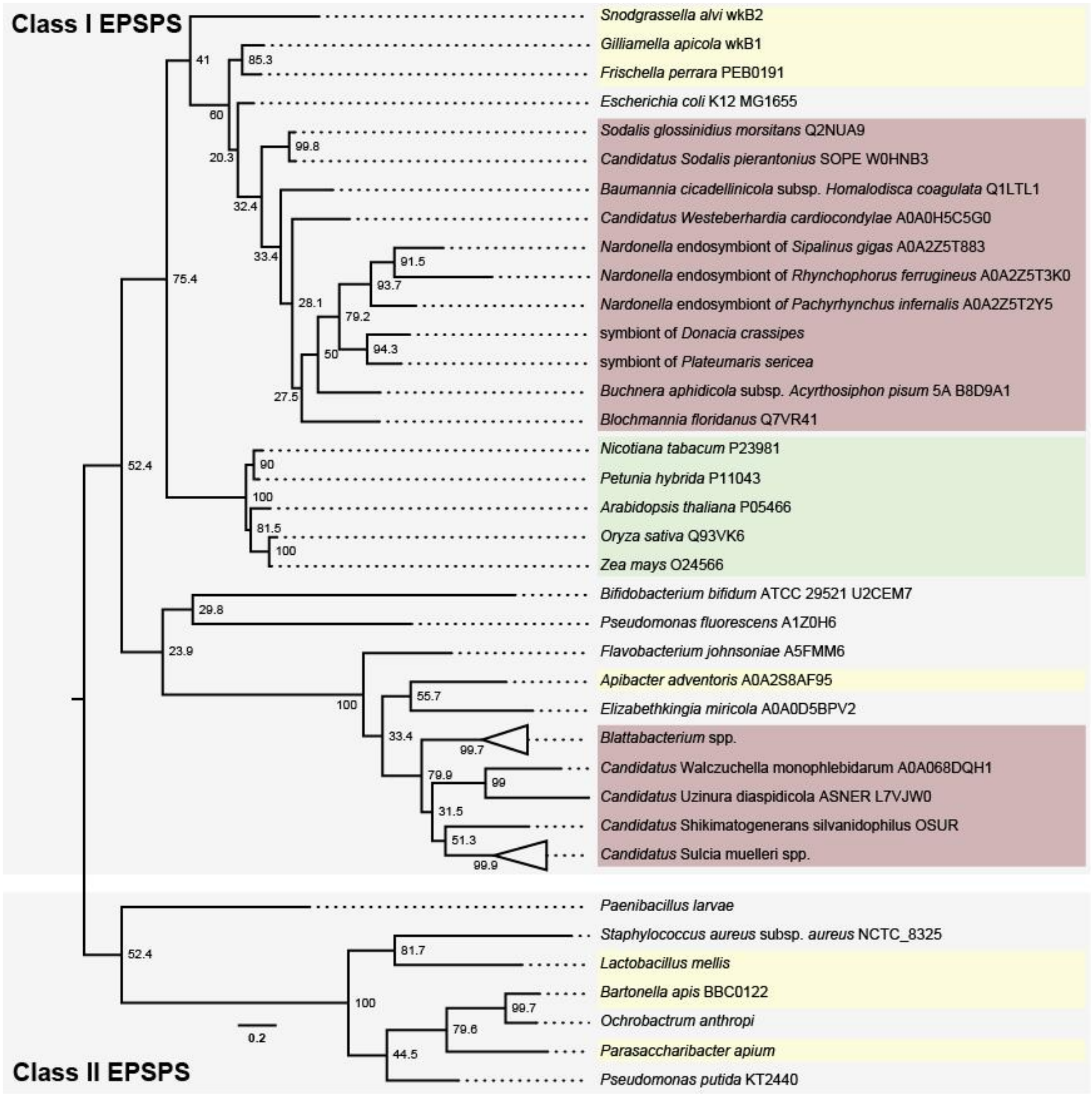

Supplementary Figure 4. Phylogenetic classification of EPSPS enzymes from different Bacteroidetes and γ-Proteobacteria insect symbionts based on PhyML and Maximum Likelihood analyses of amino acid sequences of EPSPSs using the Jones-Taylor-Thorton model and 1000 bootstrap replicates. Enzymes of plants (green), bacterial symbionts in the honeybee gut (yellow) and obligate intracellular insect symbionts (red) are highlighted. Node values indicate bootstrap values.

References

1. Aziz, R. K. et al. The RAST Server: rapid annotations using subsystems technology. *BMC Genomics* **9**, 1–15 (2008).
2. Brettin, T. et al. RASTtk: a modular and extensible implementation of the RAST algorithm for building custom annotation pipelines and annotating batches of genomes. *Sci. Rep*. **5**, 8365 (2015).
3. Overbeek, R. et al. The SEED and the Rapid Annotation of microbial genomes using Subsystems Technology (RAST). *Nucleic Acids Res*. **42**, D206-14 (2014).

4. Seemann, T. Prokka: rapid prokaryotic genome annotation. *Bioinformatics* **30**, 2068–2069 (2014).

5. Arkin, A. P. *et al.* KBase: The United States department of energy systems biology knowledgebase. *Nat. Biotechnol.* **36**, 566 (2018).

6. Parks, D. H., Imelfort, M., Skennerton, C. T., Hugenholtz, P. & Tyson, G. W. CheckM: assessing the quality of microbial genomes recovered from isolates, single cells, and metagenomes. *Genome Res.* **25**, 1043–1055 (2015).

7. Price, M. N., Dehal, P. S. & Arkin, A. P. FastTree 2–approximately maximum-likelihood trees for large alignments. *PLoS One* **5**, e9490 (2010).
