# Supplementary material for: Inhibition of a nutritional endosymbiont by glyphosate abolishes mutualistic benefit on cuticle synthesis in Oryzaephilus surinamensis
Source: Commun Biol. 2021 May 11;4:554. doi: 10.1038/s42003-021-02057-6 (PMC8113238; doi:10.1038/s42003-021-02057-6)
Supplement: Supplementary file 2 — Description of Additional Supplementary Files [file 42003_2021_2057_MOESM2_ESM.pdf]

Description of additional supplementary files

Supplementary data 1

Description: Overview results of generalized linear models on symbiont influence on relative amino acid titers in different life stages of *O. surinamensis* in worksheet one. Significant results (FDR adjusted $p < 0.05$) are highlighted in bold. Results of single generalized linear models in further worksheets.
