# Supplementary material for: Inhibition of a nutritional endosymbiont by glyphosate abolishes mutualistic benefit on cuticle synthesis in Oryzaephilus surinamensis
Source: Commun Biol. 2021 May 11;4:554. doi: 10.1038/s42003-021-02057-6 (PMC8113238; doi:10.1038/s42003-021-02057-6)
Supplement: Supplementary file 4 — Reporting Summary [file 42003_2021_2057_MOESM4_ESM.pdf]

nature research

Corresponding author(s): Tobias Engl

Last updated by author(s): Mar 8, 2021

Reporting Summary

Nature Research wishes to improve the reproducibility of the work that we publish. This form provides structure for consistency and transparency in reporting. For further information on Nature Research policies, see our Editorial Policies and the Editorial Policy Checklist.

Statistics

For all statistical analyses, confirm that the following items are present in the figure legend, table legend, main text, or Methods section.

n/a	Confirmed	
☐	☒	The exact sample size (*n*) for each experimental group/condition, given as a discrete number and unit of measurement
☐	☒	A statement on whether measurements were taken from distinct samples or whether the same sample was measured repeatedly
☐	☒	The statistical test(s) used AND whether they are one- or two-sided<br>*Only common tests should be described solely by name; describe more complex techniques in the Methods section.*
☐	☒	A description of all covariates tested
☐	☒	A description of any assumptions or corrections, such as tests of normality and adjustment for multiple comparisons
☐	☒	A full description of the statistical parameters including central tendency (e.g. means) or other basic estimates (e.g. regression coefficient) AND variation (e.g. standard deviation) or associated estimates of uncertainty (e.g. confidence intervals)
☐	☒	For null hypothesis testing, the test statistic (e.g. *F*, *t*, *r*) with confidence intervals, effect sizes, degrees of freedom and *P* value noted<br>*Give P values as exact values whenever suitable.*
☒	☐	For Bayesian analysis, information on the choice of priors and Markov chain Monte Carlo settings
☒	☐	For hierarchical and complex designs, identification of the appropriate level for tests and full reporting of outcomes
☐	☒	Estimates of effect sizes (e.g. Cohen's *d*, Pearson's *r*), indicating how they were calculated

Our web collection on statistics for biologists contains articles on many of the points above.

Software and code

Policy information about availability of computer code

Data collection	MinKNOW software (18.12.9 and 19.05.0); Qiagen Rotor-Gene Q software (v2.3.1); ZEN 2 BLue (v2.0.0.0); Natsumushi(v1.10.1); Varian Worksataion (v6.9.3)
Data analysis	SPAdes (v3.13.0); BusyBee Web (https://ccb-microbe.cs.uni-saarland.de/busybee/); RAST using the app Annotate Microbial Assembly (RAST_SDK v0.1.1) on KBase; Assess Genome Quality with CheckM - v1.0.18 in KBase; View Function Profile for Genomes (v1.4.0) in KBase; CIRCOS (v0.69-6); Insert Set of Genomes Into Species Tree v2.1.10 (SpeciesTreeBuilder v0.0.12) based on the FastTree2 algorithm in KBase; MUSCLE (v3.8.425), FastTree (v2.1.12) and PhyML (v2.2.4) implemented in Geneious Prime 2019 (2019.1.3); FigTree (v1.4.4); OrthoMCL - v2.0 in KBase; GhostKOALA (v2.2); RStudio (V 1.1.463 with R V3.6.3)

For manuscripts utilizing custom algorithms or software that are central to the research but not yet described in published literature, software must be made available to editors and reviewers. We strongly encourage code deposition in a community repository (e.g. GitHub). See the Nature Research guidelines for submitting code & software for further information.

Data

Policy information about availability of data

All manuscripts must include a data availability statement. This statement should provide the following information, where applicable:

- Accession codes, unique identifiers, or web links for publicly available datasets
- A list of figures that have associated raw data
- A description of any restrictions on data availability

Sequencing libraries and the assembles genome of the Oryzaephilus surinamensis symbiont (proposed Candidatus Shimatogenerans silvanidophilus OSUR) were uploaded to the DNA Databank of Japan (accession numbers DRA010986 and DRA010987), the NCBI Sequence Read Archive (accession numbers SRR12881563 - SRR12881566) and Genbank (accession number JADFUB000000000). Raw data of quantitative measurements are available at the data repository of the Max Planck

Society 'Edmond' under https://dx.doi.org/10.17617/3.5l.

Field-specific reporting

Please select the one below that is the best fit for your research. If you are not sure, read the appropriate sections before making your selection.

☒ Life sciences ☐ Behavioural & social sciences ☐ Ecological, evolutionary & environmental sciences

For a reference copy of the document with all sections, see nature.com/documents/nr-reporting-summary-flat.pdf

Life sciences study design

All studies must disclose on these points even when the disclosure is negative.

Sample size	Sample sizes were chosen based on estimated effect size ans sample size of a previous study: Engl et al. 2018 ncient symbiosis confers desiccation resistance to stored grain pest beetles; Molecular Ecology 27 (8): 2095-2108
Data exclusions	No data was excluded from the analyses.
Replication	In sequencing experiments are based multiple extracts/libraries generated from the same stock culture but also a different culture as well as public library. Multiple individuals or their offspring was generated in each experiment and subjected to statistical analyses.
Randomization	Individual beetles for experimental treatments were randomly picked from larger starting cultures and randomly assigned to different treatments.
Blinding	Investigators were blinded during sample analysis, meaning the group identity was not discernible from file names during analysis.

Reporting for specific materials, systems and methods

We require information from authors about some types of materials, experimental systems and methods used in many studies. Here, indicate whether each material, system or method listed is relevant to your study. If you are not sure if a list item applies to your research, read the appropriate section before selecting a response.

Materials & experimental systems

n/a	Involved in the study	
☒	☐	Antibodies
☒	☐	Eukaryotic cell lines
☒	☐	Palaeontology and archaeology
☐	☒	Animals and other organisms
☒	☐	Human research participants
☒	☐	Clinical data
☒	☐	Dual use research of concern

Methods

n/a	Involved in the study	
☒	☐	ChIP-seq
☒	☐	Flow cytometry
☒	☐	MRI-based neuroimaging

Animals and other organisms

Policy information about studies involving animals; ARRIVE guidelines recommended for reporting animal research

Laboratory animals	Oryzaephilus surinamensis strains JKI (Julius-Kuehn-Institute, Berlin, Germany) and OsNFRI (National Food Research Institute, Tsukuba, Japan)
Wild animals	The study contained no wild animals.
Field-collected samples	The study contained no field-collected animals.
Ethics oversight	No ethical approval is required for work on invertebrates in Germany.

Note that full information on the approval of the study protocol must also be provided in the manuscript.
